# The Perceptions of Elite Professional Rugby League Players and Staff on the National Rugby League Annual Calendar: A Mixed-Methods Study

**DOI:** 10.1186/s40798-023-00586-4

**Published:** 2023-06-13

**Authors:** Lewis A. Fazackerley, Geoffrey M. Minett, James D. Clark, Vincent G. Kelly

**Affiliations:** 1 School of Exercise and Nutrition Sciences, Queensland University of Technology, Kelvin Grove, QLD Australia; 2 School of Health and Behavioral Sciences, University of the Sunshine Coast, Sippy Downs, QLD Australia

**Keywords:** Football, Mental health, Physical health, Performance, Well-being, Workload

## Abstract

**Background:**

In recent years, the length of elite sporting competitions has raised concerns regarding player well-being, highlighting a need to review current match calendars. Therefore, this study aimed to explore the perceptions of elite National Rugby League (NRL) players and staff on the annual training and competition calendar from a player workload and well-being perspective.

**Methods:**

This study adopted a mixed-methods approach, using a sequential explanatory design. Phase one implemented a cross-sectional survey, and phase two utilised semi-structured interviews. Four hundred and thirty-nine elite rugby league players and 46 staff completed the survey. Eighteen elite professional NRL players and six football staff were interviewed, and verbal data were analysed into pre-defined topic summaries using qualitative coding reliability methods. Topics included in-season, off-season, pre-season and well-being.

**Results:**

Data analysis suggests that elite NRL players and staff believe players appear particularly comfortable with the current number of games; however, they are at their maximum capacity. Importantly, this study identified several minority groups that may require support to enhance player well-being. Players believe reducing the pre-season would negate fatigue experienced later in the subsequent season. Players and staff believe this timeframe still provides sufficient time to prepare for the upcoming season. Further, players were open to extending the off-season to 8–10 weeks and believed that extra time would allow for greater recovery from the previous season. Mid-season congested scheduling affects players following the intensified period and requires attention to alleviate fatigue.

**Conclusion:**

The results of this study convey important implications for the NRL, emphasising a need to review their annual training and competitive calendar, or to implement specific strategies to enhance the well-being of minority groups. The findings from this study should be considered when discussing the ideal length and structure of the match calendar to support players’ physical and mental welfare.

**Supplementary Information:**

The online version contains supplementary material available at 10.1186/s40798-023-00586-4.

## Background

The structure of sporting calendars, and the scheduling of numerous international sports competitions, have been revised to meet commercial demands [1]. For many competitions, this has increased the number of games and the frequency of intercontinental travel [2]. Such changes may increase broadcast and sponsorship opportunities for sports [3]; however, they may not always be in the best interest of player health and well-being. Gouttebarge et al. [4] reported that 40% of European footballers perceive the number of competitive games per season as negatively influencing performance and/or health. The majority of players (85%) were in favour of a 14-day in-season break and a longer off-season (> 5 weeks) [4]. However, the perceptions of elite football staff have not been explored; the opinions of whom seem pertinent in contextualising player responses should also be considered when competition schedules are re-evaluated. High-quality qualitative research is marked by the breadth of the interview sample relevant to the goals of the research questions [5]. Since staff are responsible for monitoring and managing players during the season and throughout game scheduling, their perceptions are essential to consider when investigating the annual training and competitive calendar. As such, examining the current match calendars of premier sports competitions appears necessary to ensure that they facilitate sufficient recovery, maximise player performance, and reduce injury risk [4].

The Australian National Rugby League (NRL) is one professional sports competition that has undergone marked administrative governance, implementing several scheduling and rule changes in recent years. Rugby league (RL) is continually evolving into a faster, more intensive, and more competitive game, resulting in increased physiological and perceptual workloads [6, 7]. Workload refers to the accumulation of physiological and psychological stress experienced by an athlete, resulting from training sessions and competitive games over a given period [8, 9]. Some scheduling changes, such as reducing the number of five-day turnarounds (five days between games), have been implemented to combat fatigue. In the 2016 season, there were 43 five-day breaks, which was reduced to 26 in the 2019 season [10]. However, mid-season representative games heavily interrupt the five-day break (State of Origin series). Representative games are usually played mid-week (Wednesday) between NRL premiership games, resulting in consecutive three- or four-day recovery periods and a congested schedule. This is particularly concerning, given that McLean et al. [11] suggested that measures of acute fatigue (e.g., countermovement jump and psychometrics) are reduced 48 h following a competitive RL game and may take up to 4 days to return to baseline. Further, the State of Origin is considered the most intense standard of RL worldwide [12]. As such, research suggests that mid-week State of Origin may induce fatigue that may not have dissipated before regular club fixtures on short turnarounds. In 2018, the NRL match schedule was altered by placing the second State of Origin game during a standalone weekend for the first time, allowing greater recovery time for players during the congested period. However, whether these structural changes have influenced player or staff perceptions of the match calendar concerning workload is unknown. At the forefront of the competition, player and staff perceptions should be considered when reviewing match schedules.

In addition to the extreme workloads players are exposed to, well-being in elite sport is gaining more attention [13]. The main feature of an player’s well-being is their mental health, which encapsulates a wide variety of meanings, but includes the ability to work productively and fruitfully, cope with the normal stresses of life, and when an individual is able to realise their potential [14, 15]. One major contributor to poor mental health can be overtraining, resulting from extreme training and competition workloads with inadequate rest [16]. As such, it appears pertinent to investigate mental health in elite football players, where literature in other football codes suggests players are playing too many games each season, negatively affecting their health [4]. Therefore, the opinions of players and staff on current workloads and the effect on mental health should be explored to provide a comprehensive overview of the annual training and competitive calendar. However, it is important to note that differences in perceptions of workload between players and staff/coaches have been observed in other sports, such as basketball [17, 18]. For example, ratings of observed exertion (out of 20) by staff during a game were significantly greater than the players perceived exertion (observed = 16.1 ± 1.4 vs. perceived = 15.6 ± 2.3; *p* < 0.05) [18]. These differences should be considered when interpreting conflicting perceptions of exertion by players and staff, and conclusions must be made cautiously.

When answering specific research questions, qualitative methods can complement quantitative investigations [19]. This is particularly important in exploratory research, allowing for greater versatility in discovering novel ideas offered by the qualitative approach [20]. Utilising sequential techniques, i.e., a mixed-methods approach, results in more robust validity [20]. Such holistic qualitative approaches have been used effectively in sport and exercise science research. For example, in recent years, qualitative research in football has utilised mixed-method approaches when investigating warm-up strategies [21]. Results provide valuable and reliable information on current practice in applied settings. As research on the annual training and competition calendar is scarce, conducting a cross-sectional survey, and using the results to develop semi-structured interview schedules, may provide robust data that will facilitate discussions looking to optimise the match calendar. The working definition of optimal, in the present study, will suggest that players are able to complete the season without experiencing chronic physiological or psychological stress that may have adverse effects on their overall performance and well-being. Subsequently, results may greatly benefit the applied stakeholders by balancing player workloads with game schedules.

This study aims to expand on previous cross-sectional perceptions in football and explore in detail the perceptions of NRL players and staff on the NRL annual training and match calendar using a mixed-method approach. Specifically, this study aims to explore player and staff opinions on the number of in-season games, and the optimal off-season and pre-season duration. Further, differences between player and staff responses will be investigated. Importantly, this study also aims to highlight areas of concern in relation to player workload or well-being, and key metrics that are considered important in monitoring performance, fatigue, and well-being. The findings from this study will facilitate discussions around optimising the game schedule, and providing guidelines focused on improving player well-being and welfare. Findings can be used as a framework to support guidelines that better support player’s physical and mental welfare, which is pertinent when discussing the ideal length and structure of the match calendar.

## Methods

### Study Design

This study was conducted through a mixed-methods approach, using a sequential explanatory design. Phase one implemented a cross-sectional survey design to obtain a broad understanding of the perceptions of players and staff on the annual training and competition calendar. Phase two utilised semi-structured interviews to better understand player and staff perceptions. A coding reliability approach was utilised in phase two, taking a deductive approach to thematical analysis, with pre-defined themes understood as topic summaries [22]. Topic summaries included the off-season, pre-season, in-season and well-being. Phase one occurred across the 2019 season, and phase two occurred between the 2020 and 2021 seasons. Ethics approval for this study was granted by the University Human Research Ethics Committee (phase one approval number 1900000604; phase two approval number 1900001072). This study was performed as per the standards of ethics outlined in the Declaration of Helsinki. The NRL provided written consent to allow the research team to approach players and staff to participate in both phases of this study.

### Phase One

#### Participants

Professional RL players and football staff were invited to complete a survey regarding their perceptions of the current NRL annual training and match calendar. Players and staff contracted to or employed by an NRL club participated in this study. Completion and submission of the survey were taken as an indication of consent and at no stage were participants required to provide their names, maintaining the anonymity of responses.

Four hundred and thirty-nine elite male RL players (age: 24.2 ± 3.9 years) and 46 football staff (age: 40.7 ± 6.8 years) completed phase one of the study. Based on the assumption that a full list of 36 players from 16 teams was available to participate in this study, a response rate of 76.2% (439/576) of contracted NRL players was achieved. Representatives from all 16 NRL clubs participated in this study. At the time of completion, players had played a median average (IQR) of 35 (4 to 106) NRL games (range 0 to 400) across 4 (2 to 7) seasons (range 0 to 18). The demographics of players can be viewed in Table 1. Staff included the head of performance (*n* = 12), physiotherapists (*n* = 9), committee (general managers, executive managers and officers) (*n* = 8), coaches (*n* = 6), sport science/strength and conditioning (*n* = 5), medical (*n* = 4), and well-being (*n* = 2). Staff had a median average (IQR) of 8 (2 to 12) years’ experience in their current role and 10 (7 to 16) years’ experience working in the NRL.

**Table 1:** Phase one player demographics

Marital status	*n*	%
Single	171	39.2%
De Facto	169	38.5%
Married	98	22.3%
Divorce	1	< 0.1%

Dependants		
Players with dependants	137	31.2%
If yes, how many?	*1.9* ± *1.0*	

Primary playing position		
Middle forward (8,10,13)	133	30.3%
Centre (3,4)	70	15.9%
Half (6,7)	65	14.8%
Edge back row (11,12)	63	14.4%
Hooker (9)	39	8.9%
Wing (2,5)	39	8.9%
Full-back (1)	30	6.8%

Experience		
International	151	34.4%
Development squad	65	14.8%
State of Origin	60	13.7%

International Representative Nation		
Australia	31	20.5%
New Zealand	30	19.9%
Samoa	19	12.6%
Fiji	14	9.8%
Tonga	12	7.6%
*Other*	45	29.6%

Dependants include children only (where those who had dependants were asked how many [presented as mean ± SD]). Playing position includes the standardised number, which reflects their role in attack and defence. Nations with a representation < 5% are listed as other. Other includes England, Lebanon, New Zealand/Samoa, New Zealand/Tonga, Cook Islands, PNG, Australia/Tonga, Italy, New Zealand/Cook Islands, Samoa/Tonga, Australia/Samoa, Canada, Malta, and Scotland.

#### Survey Design

This observational study was based on a cross-sectional design using a survey (Additional file 1). The researchers employed a Delphi validity approach [23] to develop and refine questions based on previous cross-sectional research investigating the competitive calendar in Elite Football [4]. To refine the survey, expert meetings with NRL and Rugby League Players Association (RLPA) representatives were held to refine questions and statements. The survey went through three rounds of feedback to reach a consensus on the final survey design.

The survey was implemented using KeySurvey (Version 8.26, WorldApp, Braintree, Massachusetts) and pilot-tested with a small sample of academics, practitioners, rugby league football and administrations staff, and NRL research committee members (*n* = 10) to establish its clarity and feasibility. Following approval by the NRL, a secure URL link was distributed to participants during a team meeting at respective club facilities and completed individually. If participants could not access the online survey (*n* = 44), a paper-based survey was provided and handed to the research team immediately following completion.

The survey took approximately 10 min to complete. Surveys were completed during an 8-week period, corresponding to Round 18 to Round 24 of the NRL match calendar. Participants were required to complete between 11 and 16 questions, with only representative players required to complete all questions. Staff were asked the same questions as players, albeit re-worded in the third person. For example, ‘I am playing too many games per season’ (player question) was presented as ‘athletes are playing too many games per season’ (staff question).

The first component of the survey obtained demographic information. For players, this included age, marital status, dependants, primary playing position, the total number of first-grade NRL games and seasons completed, and whether participants had participated in mid-season or end-of-season external representative games within the last five years (i.e., State of Origin or international games). Staff demographics included age, sex, staff role, years of experience in the role, and years of experience within the NRL. The second component of the survey included questions and statements relating to the number of games played each year and the associated effect on performance, and physical and mental health. Additionally, opinions on the off-season and pre-season periods were explored. Questions and statements were either scored on a 4-point Likert scale; ‘Strongly Disagree, Disagree, Agree, Strongly Agree’, or a blank space was provided for participants to enter a single number representing an amount in weeks. A neutral Likert scale response was omitted from the survey to encourage respondents to formulate an opinion and avoid satisficing [24].

#### Statistical Analysis

Data were exported from KeySurvey to Microsoft Excel (2016, Microsoft, Redmond, WA, USA), with each response being assigned a non-identifying number. Numerical values were assessed for normality using a Shapiro–Wilk test. Chi-square test (categorical data) or Kruskal–Wallis test (numerical data) assessed whether the week of completion influenced responses. Adjusted residuals were calculated to identify significant contributing cells or categories in the Chi-squared analysis. Epsilon-squared were used to assess the magnitude of differences in the Kruskal–Wallis analysis (Epsilon ES). For this, Likert scale responses were collapsed into two categories: agree and disagree, to ensure all cells had > 5 responses. Chi-square linear trend analysis assessed the distribution of significant responses. Group differences between players and staff were calculated using Pearson’s Chi-squared tests (categorical data) or independent *t* tests (numerical data). If the Chi-square test assumptions were violated, Fisher’s exact test was used. Player sub-category differences were calculated using Chi-square (categorical data) or a Kruskal–Wallis test (numerical responses). In the case of significance, Kruskal–Wallis multiple comparisons examined the source. For sub-category differences, Likert scale responses were collapsed into ‘agree’ and ‘disagree’. Sub-categories included player level (no representative duties, State of Origin/international duties, State of Origin/no international duties, no State of Origin/international duties, and development), family status (single/no dependants, single/with dependants, de facto or married/no dependants, de facto or married/with dependants), and playing position (middle forward, centre, half, edge back row, hooker, wing, full-back). Only one player reported being divorced and was removed from the family status subgroup analysis. Numerical variables are presented as mean ± standard deviation, and categorical variables are presented as a proportion (*n*, %) of responses. Statistical analyses were performed using GraphPad Prism 8 for Windows 64-bit (version 8.2.1; GraphPad Software, La Jolla, CA, USA). The alpha was set at 0.05.

### Phase Two

#### Participants

An online invitation and consent form to participate in this study was distributed to all NRL players and various staff departments of NRL clubs. The invitation was also distributed via respective club communication channels by RLPA representatives. Thirty-three players and six staff volunteered to participate. To provide a representation of players across the league, players were categorised per their experience: limited (2–3 seasons, minimum 25 games), moderate (4–5 seasons, minimum 50 games), and considerable (6 + seasons, minimum 100 games). First-year players were omitted due to insufficient time spent in the NRL system. Six players from each experience group (*n* = 18) were selected using an online random generator (https://randomwordgenerator.com/name.php) and interviewed. If a player withdrew before the interview, another player was randomly selected from the same group. Only staff (*n* = 6) working within the multidisciplinary sport science team at an NRL club were considered. Semi-structured interviews were conducted using Zoom software (Zoom Video Communications Inc., San Jose, California). Subsequently, a professional transcription company saved the audio as a non-identifiable audio-only file (M4A) for verbatim transcription. To ensure accuracy in the transcriptions, files were reviewed by the lead author.

Players included six with limited experience, six with moderate experience, and six with high experience (randomised as Player 1 to 18) and were from 10 different NRL clubs. Staff (randomised as Staff 1 to 6) represented 6 different clubs and included Head of performance (*n* = 3), strength and conditioning (*n* = 2), and a head physiotherapist (*n* = 1). Staff had spent 13.7 ± 4.0 years working in the NRL. The average recorded interview duration with players was 29.8 ± 7.4 min and 36.5 ± 5.0 min for staff. In this results section, four themes are presented (in-season, off-season, pre-season and well-being), with a total of 11 sub-themes reflecting how players and staff perceive the annual training and match calendar.

#### Topics of Discussion

To establish face validity, semi-structured interview questions were developed based on the results from phase one. To ensure balance in the interview schedule, and to ensure findings would benefit the stakeholders, questions and statements were reviewed by the NRL and RLPA. The NRL and RLPA provided feedback on the interview schedule, and the research team discussed in detail whether the change would be accepted or rejected. Guided questions focused on the optimal length of the off-season, pre-season, and competitive season periods. An abbreviated interview schedule can be viewed in Table 2, and the full interview schedule as Additional file 2. Within these dimensions, probing questions addressed important considerations, current challenges, positive and negative factors, and future considerations.

**Table 2:** Abbreviated interview schedule (without probing questions) on the off-season, pre-season and in-season components of the annual training and match calendar and players well-being

Season component	Question
Off-season	Q1. What do you think is the optimal period for the off-season?
Pre-season	Q2. What do you think is the optimal period for the pre-season?
In-season	Q3. Typically, how many games should NRL players be playing every season (both club and representative levels)?
	Q4. What changes, if any, should be made in game scheduling?
	Q5. For representative athletes, how do you think representative games affect you?
	*Q6. What factors contribute to player fatigue during the season?
	*Q7. What factors contribute to player performance levels during the season?
	*Q8. How does the club currently monitor player workloads during the season?
	*Q9. What changes (if any) need to occur to improve the process of managing player fatigue/maintaining player performance levels in-season?
Well-being	*Q10. How does the club currently monitor player well-being during the season?
	*Q11. What factors impact player well-being (physical/mental/emotional health) during the season?

*Questions 10 and 11 were directed towards players, and questions 6 to 9 were directed at staff

Immediately prior to the interview, a standardised pre-amble was read verbatim to all participants, which provided clear instructions on the interview process and allowed participants to seek clarification. This ensured that all participants had a consistent comprehension of the interview process and could provide comparable responses. Additionally, precautions were taken to ensure participants were alone in a quiet area, to minimise external disturbance. Participants were requested to turn on their camera (without recording), to allow the interviewer to confirm that the participant was in a suitable environment.

#### Data Analysis

The philosophical orientation of the research team was positivist, which influenced the scientific analysis of the data [25]. Initially, the lead author immersed themselves in the data by reading and re-reading the audio recording’ verbatim transcripts. Following this, a codebook was devised and applied to the entire raw data set, taking a deductive approach to thematical analysis [26], using qualitative data analysis software (NVivo 12, QSR International, Australia). A random sample of transcripts (*n* = 4 transcripts, 16.7% of dataset) was provided to a colleague with experience in qualitative research to determine intercoder reliability as recommended for good practice [27], which aligns with the positivist paradigm [25]. The colleague was not involved with the study design and was familiarised with the codebook by the lead author. Reliability between coders for each transcript was calculated using Cohen’s Kappa index, obtaining values up to 0.71 for inter-coder reliability over 138 interview responses, considered substantial and acceptable [27]. The coded data were then inductively analysed and sorted and amalgamated into broader themes. Due to the semi-structured nature of the interview schedule, the concept of data saturation was avoided [28]. Themes were summarised, with the proportion of players (*n*) who contributed to the themes identified. Findings were visualised as word clouds to display the frequency of identified factors using online software (https://wordart.com/create).

## Results

### Phase One

#### Player and Staff Responses

**Fig. 1:**
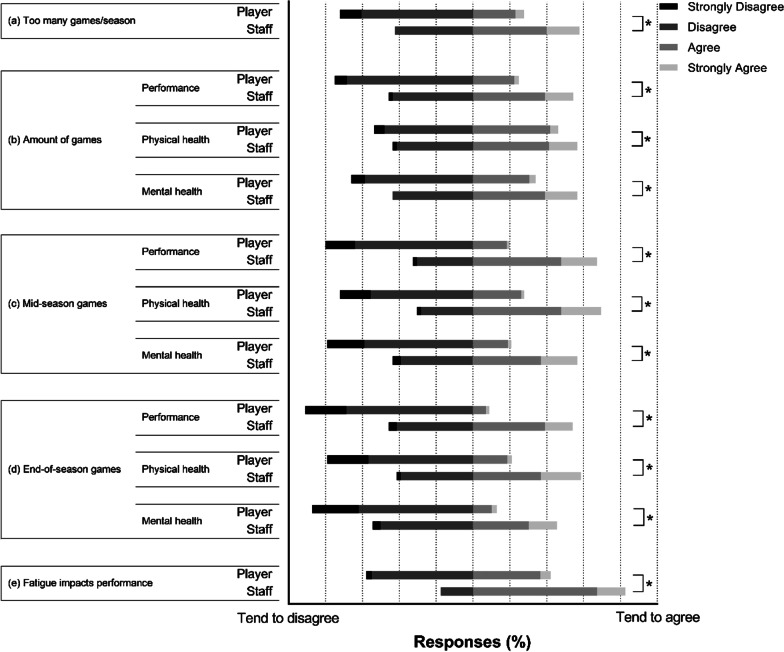
Player and staff perceptions of the in-season period. In-season Likert scale responses on the number of games played (**a**, **b**), and the effect of mid-season (**c**) and end-of-season (**d**) representative duties, and whether fatigue affects performance (**e**). Questions/statements are summarised. Each bar segment represents a percentage of players or staff and their perception of each question/statement (strongly disagree to strongly agree). Bars sitting further to the left tend to disagree, and bars sitting further to the right tend to agree. *denotes a significant difference (*p* < 0.05) in responses between players and staff for the same question/statement

Figure 1 shows player and staff responses to in-season questions/statements on performance, fatigue and physical and mental health. Opinions of players and staff contrasted one another concerning the number of games played (*χ*^2^ = 23.80, *df* = 3, *p* < 0.001), and the effect of the number of games on performance (*χ*^2^ = 24.99, *df* = 3, *p* < 0.001), physical health (*χ*^2^ = 10.29, *df* = 3, *p* = 0.0162), and mental health (*χ*^2^ = 23.99, *df* = 3, *p* < 0.001). Differences between representative players and staff perceptions were observed regarding representative games having negative consequences on performance (mid-season: *χ*^2^ = 10.29, *df* = 3 [*p* = 0.0162], end-of-season: *χ*^2^ = 48.73, *df* = 3 [*p* < 0.001]), physical health (mid-season: *χ*^2^ = 23.99, *df* = 3 [*p* < 0.001], end-of-season: *χ*^2^ = 34.57, *df* = 3 [*p* < 0.001]), and mental health (mid-season: *χ*^2^ = 23.99, *df* = 3 [*p* < 0.001], end-of-season: *χ*^2^ = 28.35, *df* = 3 [*p* < 0.001]). There were no identified trends or linear associations between the round of completion and categorical (*χ*^2^ range = 0.65 to 2.76, *df* = 3, *p* range = 0.398 to 0.884, adjusted residual range = 0.17 to 0.33) or numerical responses (*χ*^2^ range = 1.13 to 3.20, *df* = 7, *p* range = 0.784 to 0.980, Epsilon ES range = 0.01 to 0.02).

Forty-two percent of players believed they experience fatigue that negatively impacts performance, whereas 83% of staff agreed. (*χ*^2^ = 29.14, *df* = 3, *p* < 0.001). Of those who agreed, players (*n* = 183) believed the fatigue occurred during Round 15 (of 24) and lasted 3.9 ± 3.0 weeks. Staff (*n* = 38) believe it occurred during Round 14, lasting 4.5 ± 1.7 weeks.

**Fig. 2:**
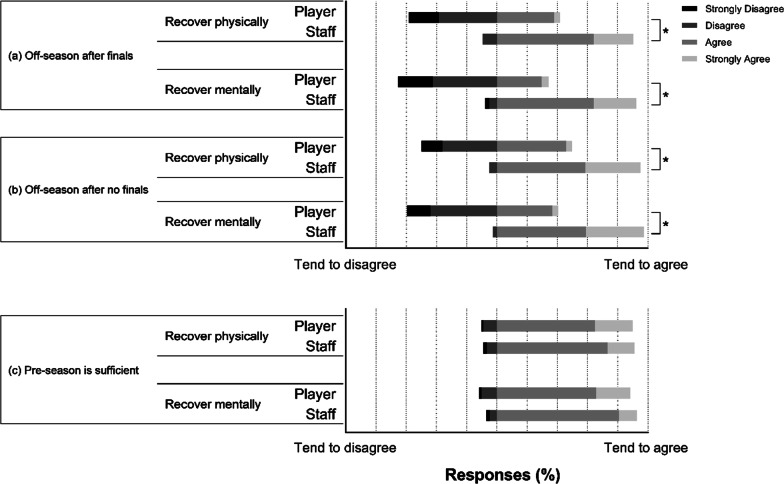
Player and staff perceptions of the off-season and pre-season period. Off-season and pre-season Likert scale responses on whether the length of the off-season period, whether they do (**a**) or do not (**b**) participate in the NRL finals series, is sufficient to recover physically and mentally, and whether the pre-season is sufficient to prepare physically and mentally (**c**) Questions/statements are summarised. Each bar segment represents a percentage of players or staff and their perception of each question/statement (strongly disagree to strongly agree). Bars sitting further to the left tend to disagree, and bars sitting further to the right tend to agree. *denotes a significant difference (*p* < 0.05) in responses between players and staff for the same question/statement

Figure 2 illustrates player and staff responses to the off-season and pre-season question/statements on physical and mental recovery and preparation. When players are involved in finals games, player and staff perceptions contrast on the length of the off-season being sufficient to recover physically (*χ*^2^ = 45.43, *df* = 3, *p* < 0.001) and mentally (*χ*^2^ = 51.47, *df* = 3, *p* < 0.001). Similarly, when players do not play finals, the period was sufficient to recover physically (*χ*^2^ = 71.43, *df* = 3, *p* < 0.001) and mentally (*χ*^2^ = 77.22, *df* = 3, *p* < 0.001). No differences were observed between player’s and staff’s perceptions of the length of the pre-season being sufficient to prepare for an upcoming season, physically and mentally. Perceptions on the length of the off-season and pre-season period are shown in Table 3.

**Table 3:** Player and staff perceptions on the length of the off-season and pre-season period to recover and prepare, respectively. Answers are in weeks

	Player	Staff	*p* value
*Off-season*	*n* = 439	*n* = 44	
Complete inactivity	3.1 ± 2.0	–	
Optimal length	9.0 ± 3.8*	7.3 ± 1.4*	0.005

*Pre-season*			
Optimal length	9.2 ± 3.1*	11.5 ± 2.9*	< 0.001
Minimal length	7.3 ± 2.8*	9.2 ± 2.7*	< 0.001

*Denotes answers significantly different from one another within each row (*p* < 0.05)

#### Player Sub-Categories

There was no effect of playing position on responses. The effect of family status and player level on responses to questions relating to the length of the off-season and in-season period can be viewed in Tables 4 and 5.

**Table 4:** Family status and response to questions regarding the off-season and pre-season period. Answers are in weeks

	Single/no dependants	Single/dependants	De facto/married/no dependants	De facto/married/ dependants
*Off-season*	*n* = 164	*n* = 7	*n* = 138	*n* = 129
Complete inactivity	2.99 ± 1.89	2.57 ± 1.27	2.93 ± 2.09*	3.46 ± 2.04*
Optimal length	8.45 ± 1.95*	8.86 ± 3.08	8.78 ± 1.72	9.22 ± 1.98*

*Pre-season*				
Optimal length	9.80 ± 3.25	9.29 ± 3.77	8.87 ± 2.67	8.75 ± 3.11
Minimal length	7.76 ± 3.18*	8.00 ± 2.83	7.14 ± 2.46	6.71 ± 2.49*

*Denotes answers significantly different from one another within each row (*p* < 0.05)

**Table 5:** Player level and response to questions regarding the off-season and pre-season period. Answers are in weeks

*Off-season*	*n* = 203	*n* = 35	*n* = 25	*n* = 111	*n* = 65
Complete inactivity	2.86 ± 1.84*	4.03 ± 2.43*	3.29 ± 1.27	3.38 ± 2.36	2.81 ± 1.64
Optimal length	8.59 ± 1.57*	9.17 ± 2.53	9.60 ± 1.44*,**	9.18 ± 2.06***	8.12 ± 2.23**,***

*Pre-season*					
Optimal length	9.50 ± 2.83*	8.80 ± 2.44	7.92 ± 2.53*,**	8.30 ± 2.94***	10.46 ± 3.90**,***
Minimal length	7.37 ± 2.72	7.00 ± 2.65	6.16 ± 1.60*	6.57 ± 2.66**	8.68 ± 3.07*,**

*,**,***Denote answers which are significantly different from one another within each row (*p* < 0.05). SOO = State of Origin. Int. rep = International representative duties. Dev = Development player

**Fig. 3:**
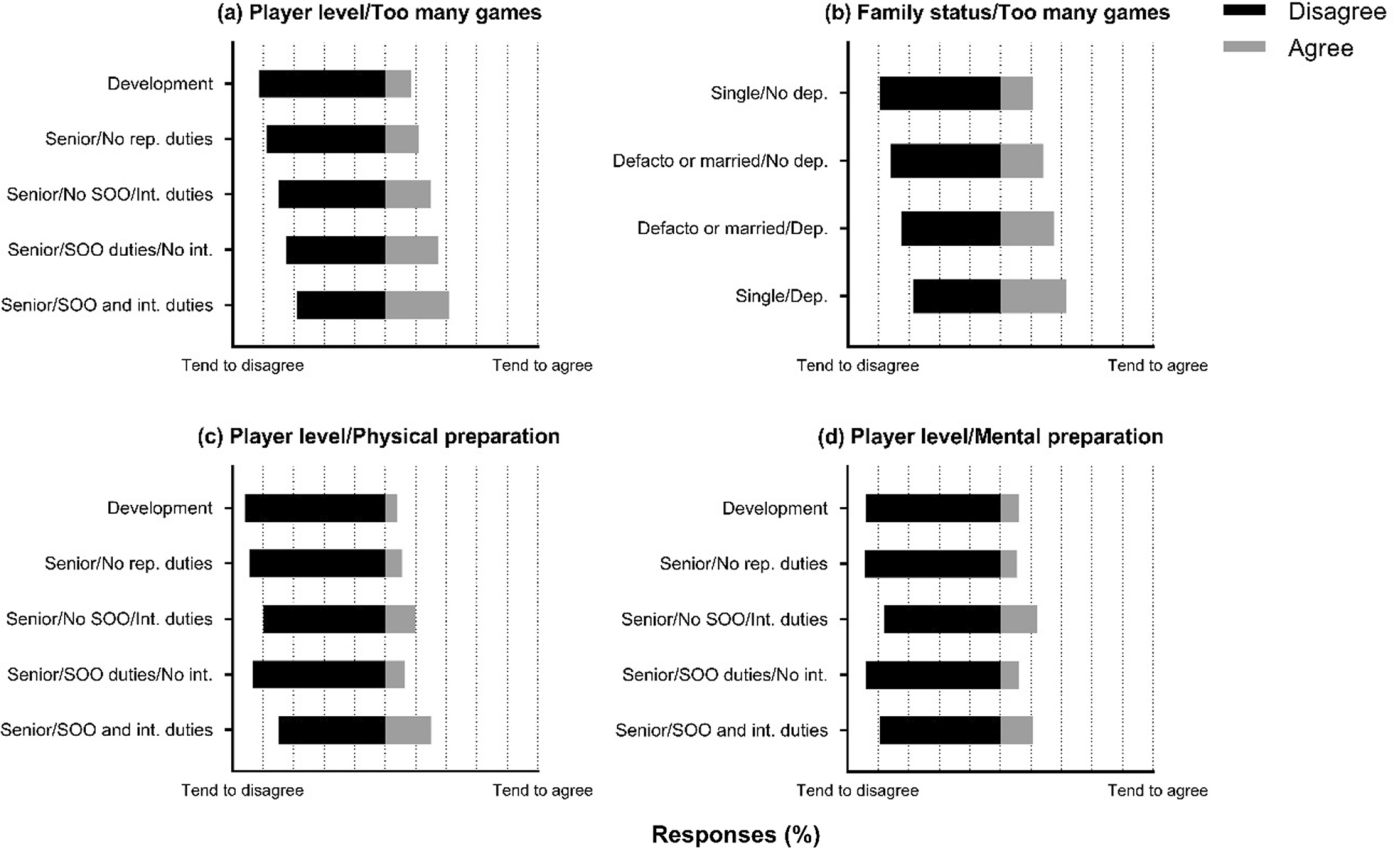
Influence of player level (**a**) and family status (**b**) on perceptions of playing too many games each season, and player level perceptions on the pre-season period being sufficient to prepare physically (**c**) and mentally (**d**). SOO = State of Origin. Int = international duties. Dep = dependants

Figure 3 illustrates the influence of family status (*χ*^2^ = 8.00, *df* = 3, *p* = 0.046, adjusted residual range = 3.1 to 3.8) and player level (*χ*^2^ = 10.16, *df* = 4, *p* = 0.038, adjusted residual range = 3.4 to 4.2) on perceptions of playing too many games each season. Family status also influenced responses on the influence of the number of games played on mental health (*χ*^2^ = 8.282 *df* = 3, *p* = 0.0405, adjusted residual range = 3.5 to 4.7). Fifty-seven percent of players who were single with dependants and 41% who were de facto or married with dependants agreed that the number of games negatively affected their mental health. Sixty-six percent of de facto or married players without dependants disagreed, and 74% of single players without dependants disagreed. Figure 3 also illustrates the influence of player level on whether the pre-season period is sufficient to prepare for an upcoming season physically (*χ*^2^ = 11.84, *df* = 4, *p* = 0.0186) and mentally (*χ*^2^ = 10.27, *df* = 4, *p* = 0.0360). Player level influenced responses on the off-season period being sufficient, when not participating in finals, to recover mentally (*χ*^2^ = 9.596, *df* = 4, *p* = 0.0478). Fifty-seven percent of development players and 46% of State of Origin players who do not represent their nation agreed that the period is sufficient. Compared to 41% of senior players who do not play representative games, 41% of senior players who played State of Origin and do not represent their nation, and 27% of players who do not play State of Origin yet partake in representative duties.

### Phase Two

#### In-Season

##### Length

Players (*n* = 13) stated they were either happy with the current number of games being played each season or were open to reducing the number (*n* = 4). Generally, 20- to 24-games were preferable.

*But overall, I think you can easily get out 24 games in a season. I don't think it's too long at all* – **Player 7 (medium experience)**

Staff responses were similar to players, who agreed with the current number of games (*n* = 4), with some leaning towards reducing the number of games (*n* = 2). Like players, staff believe some struggle with injury towards the season’s end (*n* = 3).

*I don't think there's too much wrong with it, but I do think we're pushing the upper limit of how much football they can play in a year* – **Staff 2**

*What they're doing right now, it's too much. I’ve worked with teams in two grand finals, and got to six final series. By that stage, they’re hanging on*. *I think we can probably get more use of the players if we didn't have such long seasons. If money wasn't an issue, I'd sort of be saying anywhere between 20 to 23 rounds* – **Staff 4**

##### Impact of the Current Structure

Players mostly disagreed that the current number of games negatively impacts performance (*n* = 10), and physical or mental health, when they are fit. Some players (*n* = 5) also suggested that players often play through an injury and that performance may diminish during prolonged seasons. However, some players perceive performance and physical health to diminish, but it was not of concern (*n* = 3).

*It's all good as long as you're healthy. It's when you get a little injury that you've got to keep playing with* – **Player 1 (low experience)**

*I think that the NRL would get the greatest benefit from shortening up the season, because a lot of players do play with niggly injuries, which affects their performance and they're riddled throughout every club. If that's affecting performance, it's going to affect the product of the game* – **Player 14 (high experience)**

Staff believe that the number of games takes a mental toll on players, particularly during mid-year representative duties (State of Origin and Internationals) and that teams suffer from fatigue when representative players return to their club (*n* = 4).

*Physical health, I think they’re okay with it, mental health, I have seen the effects on a number of players in our club being worn down by the number of games that they've played* – **Staff 2**

*Some teams suffer with post Origin fatigue* – **Staff 4**

*No doubt, some teams suffer with that post Origin fatigue* – **Staff 6**

##### Game scheduling

Mid-year representative players (State of Origin or International) (*n* = 6 of 9 representative players) find it physically and mentally challenging to back-up mid-week representative games with club games 2 to 4 days later. However, recovery significantly improves when playing representative games on a standalone weekend.

*I think we had a six day turn around into the next club game. And yeah, that’s enough time to recover and get ready and switch over to club mode. And then when you’re playing two or three days later, you’re just still cooked. I think allowing people to have some good recovery time is probably a good option* – **Player 13 (high experience)**

Regarding the game schedule, players (*n* = 7) appreciated the recent reductions in 5-day turnarounds. Mostly, players believed they could recover week-to-week with the rare 5-day turnaround. However, some players would like to see them removed completely (*n* = 5).

*I think turnaround times aren’t too bad. I know they’ve done a lot of work to try and make it fair and everything. And probably shouldn’t compare, but I speak to people that I know who play overseas in England and that, and they play two or three days later, they play two games within four days sort of thing. And I think that’s just crazy* – **Player 2 (low experience)**

*It’s extremely tough to get the inflammatory response out of your body after playing an NRL game, to then turn around and play five days later* – **Player 8 (medium experience)**

Staff preferred the removal of all 5-day turnarounds (*n* = 4), suggesting that the minimum turnaround should be six days. Further, some staff were concerned about the fairness of turnarounds between teams (*n* = 2).

*I think a six day turn out for rugby league is the shortest that you should have with the amount of contact where, we talked about getting bigger, faster and stronger and those kind of things* – **Staff 1**

*I think five-day turnarounds need to go, or limit it to one per club per year. We’ve had a lot of five-day turnarounds with travel. A five-day turnaround with travel is just, it’s bloody hard from a scheduling and a planning point of view* – **Staff 6**

*They [another team] only had four games this year where they played an opposition who had a longer turnaround than them… So they either had the same preparation or more preparation for 20 of their games out of 24, whereas we had 13 games that our opposition had longer turnarounds than us* – **Staff 5**

Including an extra bye round (weekend off) was popular (*n* = 6). Most players who suggested they would like to have a week off playing approximately one-third and two-thirds of the way throughout the season.

*You could give blokes an extra rest during the year, and then an extra week’s rest after each season. I think you’d see great growth in the players’ wellbeing and also their player performance, because you’d be able to keep the better players playing for longer* – **Player 8 (medium experience)**

The idea of another bye was popular with staff too (*n* = 4), especially for the State of Origin players.

*Two rest periods throughout the year’s okay. But like I said, the Origin boys that don’t get it. So maybe you do have to go back to standalone rep weekend plus two byes* – **Staff 1**

#### Off-season

##### Length

The majority of players were in favour of extending the off-season period (*n* = 13). Typically, 8- to 10 weeks was the most common preferred length. Players believed the extension was required for mental rather than physical recovery (*n* = 8).

*Just being able to get away from that environment, and not think about footy, and the pressures of footy, and not have to worry about, this week, how have I performed? Or what do they think of me? Or am I going to make the team? It can be pretty draining* – **Player 8 (medium experience)**

*Somewhere between eight and 10, depending on the level. I don't know if it [6 weeks] was really long enough, you know, like mentally* – **Player 6 (low experience)**

Staff mostly agreed that eight weeks would be beneficial for players to recover, specifically mentally (*n* = 4). However, performance staff are primarily concerned about having enough time to prepare players in the pre-season and work backwards from these time frames (*n* = 3).

*I'd probably say about the eight weeks. Just sufficient amount of time for them to get away from the intensities of training, and playing, and scrutiny that is professional rugby league. We scare them into coming back in good shape and I've always wondered if we weren't as aggressive on that, would they come back a little bit more mentally refreshed?* – **Staff 2**

*For me, it's, it's not so much how much time is between end [of the season] and start of training, it's more, how long do we have to prep them for games?* – **Staff 5**

##### Recovery

Some players suggested that they can recover both physically and mentally and are ready to return within the currently prescribed off-season (*n* = 5). However, other players believed they suffer towards the season’s end (*n* = 8), which could be negated by extending the off-season to allow for greater recovery.

*You come back fresh at the start. But by the end of the season, you’re starting to drain, you’re starting to feel the effects* – **Player 7 (medium experience)**

##### Structure

Setting off-season physical targets (e.g., skin folds, running times, strength levels) was raised, and if players were on track to achieve them, they suggested the off-season should be extended (*n* = 5). Players suggested this was a beneficial way to reward those who train during the off-season. Staff also mentioned that having more control during the off-season and providing some off-season targets for players to achieve is important (*n* = 3).

*Here's your goals you have to meet on day one. We're going to meet, say at the end of the seven weeks holidays, eight weeks holidays. We'll see where you are in terms of what we've left you. If you're close, we'll see again in another couple of weeks. If not, maybe start to come in and have some unstructured training, 2-3 days a week to try to get to where we want you, so when the team's all back, we're all at a similar level* – **Player 2 (low experience)**

*I think being able to mandate certain minimum requirements they have to do in the off-season, I think's important* – **Staff 6 (medium experience)**

However, players knew the challenges this could cause some of their less experienced and/or motivated peers.

*I can see it being a problem. There's a lot of guys that would have issues physically, probably just weight-wise. Because some of them need a little bit more motivation to train, or they need to train with someone, or have someone telling them what to do to stay in shape* – **Player 16 (high experience)**

Some staff (*n* = 2) believe a critical consideration that should be explored is players who have surgery immediately following the season, who have to complete rehab and then return to pre-season without time off.

*they're having surgery as soon as the season's over, and they're in a sling or on crutches, they're not really getting time away whether it's with their family or with their mates. they can't be forced to come in, but it's in their best interest to come in and do their rehab. So they're effectively not really getting an off-season* –** Staff 6**

#### Pre-season

##### Length

The majority of players were in favour of reducing the pre-season period (*n* = 16). Generally, the most common preferred length was 10- to 12 weeks in total, including trial/practice games. As such, they mostly suggested that pre-season start in early December.

*Somewhere between three and four weeks before Christmas, … then it would probably be four to six weeks after, and then maybe a couple of practice games. So yeah, that would work out to be somewhere between 10 and 12 weeks, I would say* – **Player 13 (high experience)**

*Eight weeks before Christmas and then having a couple weeks off and then coming back to do another 10 months straight, it’s pretty hard* – **Player 10 (medium experience)**

Staff agreed that returning 4 to 5 weeks before Christmas would allow for adequate preparation (*n* = 5).

*I think four weeks before Christmas is enough generally because then you’ve got another six, eight weeks, January, February. So there’s 12, you still got your Christmas break* – **Staff 1**

##### Structure

The idea of staggered starting dates based on experiences (seasons in the NRL system) was popular among players, along with the idea of a progressive return in training workload (*n* = 7).

*I think they could almost do it so that there's a ramp. When training starts say three days a week to start. And then eventually you build to five days a week* – **Player 1 (low experience)**

Staff also agreed that staggered starting dates are beneficial based on experience (less experienced players return early) and that a gradual return to training could be beneficial (*n* = 4).

*I think getting players back into part-time training could be a good option. Start by training Monday, Wednesday, Friday. Hammering and flogging guys from day one, I think is a bit of an issue in Rugby League* – **Staff 6**

##### Preparation

Players mostly believed that ‘peak pre-season fitness’ is achieved well before trial/practice games (*n* = 10), and from then on, pre-season becomes a mental battle (*n* = 6). Some players suggested that players start to break down after this point physically.

*I reckon 16 weeks is too long. Particularly the teams that don’t make finals, this year’s an exception, but if you don’t make the finals, you generally do seven weeks pre-Christmas. Every club I’ve been at, even the young kids, a month in, by the first week of December, physically, everyone’s flying* – **Player 16 (high experience)**

*Are we getting ourselves to the point where we’re sort of like might even break down?* – **Player 18 (high experience)**

Staff agreed that peak fitness is achieved in January (*n* = 3).

*Yeah, probably not till January though [peak fitness], but that’s probably the style of training we do. If you’ve got a match fit in December, you’ve done something wrong* – **Staff 1**

Some players believe that ‘match fitness’ does not occur until a couple of games have been played and that trial games are crucial (*n* = 4).

*No matter how long and hard I train for it over the pre-season, I always find you always blow out pretty quick in those first couple of trial games. I think most players would agree on that. That’s probably the most important thing getting ready to play a season game, is those couple of trial games* – **Player 14 (high experience)**

*I don’t know how much match fitness I can get just from training, I need to play games to get my match fitness* – **Player 6 (low experience)**

##### Contact

A pre-season issue raised by a number players was the amount of contact or collision-based training (*n* = 8). These players appeared concerned about the effects on their physical health and the longevity of their careers.

*I think that the contact one is a bit of an issue. I think three or four contact sessions [per week] in the preseason is excessive, it's just too much* – **Player 16 (high experience)**

*And I'm speaking from a player welfare side, I want players to have a long career, the longest career they can have. The more they do those contact sessions, man, it's just chipping away at your body, slowly chipping away* – **Player 15 (high experience)**

Players understood why contact was being prescribed (to condition them for the season), but they suggested reducing the amount of contact (*n* = 8). The idea of progressively increasing contact over the pre-season was suggested (*n* = 3).

*If you had it [contact] from January, you don't start until for the second week of March, I think the season starts. So you've still got eight to 10 weeks of solid training that you can do with contact work* – **Player 8 (medium experience)**

##### Freshness

Players had mixed responses regarding feeling fresh physically and mentally towards the end of the preseason. Mostly, players feel good mentally as they have completed the pre-season and are about to start playing football (*n* = 7), and physically, they feel better as training tapers down (*n* = 7). However, some players are feeling the effects of a prolonged pre-season period (*n* = 6).

*Mentally, you feel good because pre-season is over* – **Player 17 (high experience)**

*Physically most of the time they're pretty good at tapering you about two weeks out to feel physically ready* – **Player 1 (low experience)**

*Sometimes, when the pre-seasons feels long, it takes you a while to get into the first half of the season because you're still kind of feeling a bit run down* – **Player 7 (medium experience)**

#### Player well-being

There were mixed responses concerning player well-being. Some players thought it needed improving at their club (*n* = 7), and others thought their club was doing a good job (*n* = 7). Responses suggested that players enjoyed having personal contact with a well-being officer and respected having a good relationship with them rather than using a well-being app (*n* = 6).

*Because you just tell on a laptop if you feel good or not. I think they probably need to do more one-on-one checks* – **Player 1 (low experience)**

*I think just getting around and asking blokes how they were going personally is, would be a good start* – **Player 13 (high experience)**

Staff supported the idea of players having someone outside of the high-performance team to speak to regarding their welfare was important (*n* = 3).

*One thing I know we've done well at [CLUB NAME] this year is we've invested a lot more in welfare. A full-time welfare officer and staff around [THEM], which I think has been an extra voice or a place to go to if they [athletes] have any issues that they might not be willing to voice to [PERFORMANCE STAFF]* –** Staff 6**

Some players were unsure how their well-being data was being used and whether it was a box-ticking exercise (*n* = 3).

*I reckon they've done it pretty good, but it was just a level that was it because that's the numbers that you need to crunch? Or, were we actually doing it for the wellbeing of the players? Probably both without sounding like a cynic* – **Player 2 (low experience)**

*Yeah. I don't know exactly how they use it all, but I think with the app, you've really got to flag something on there for them to actually come to you and talk to you about it* – **Player 13 (high experience)**

However, some players suggested the onus is on themselves, and being honest, for a successful well-being program (*n* = 4).

*They're pretty on top of it, as long as you're being honest, I think it's a pretty good system* – **Player 6 (low experience)**

**Fig. 4:**
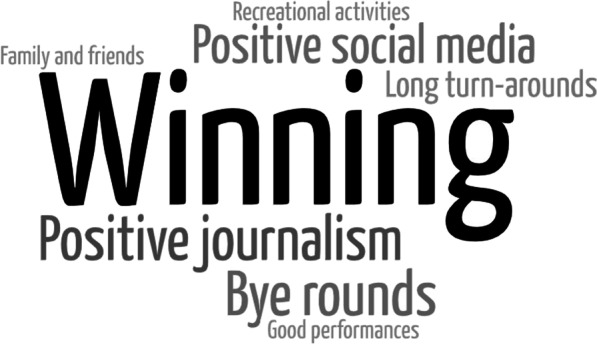
Word cloud presenting the frequency of factors that players suggest positively influence their physical, mental and emotional well-being

**Fig. 5:**
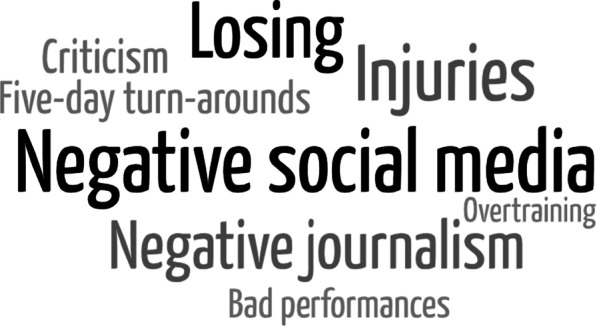
Word cloud presenting the frequency of factors that players suggest negatively influence their physical, mental and emotional well-being

Players identified various factors that positively or negatively influence their well-being (Figs. 4, 5). Players understood that winning and losing are inherent. However, the game outcome was a major factor contributing to mental well-being. Further, individual performance (good or bad), social media (positive or negative), journalism/social media (positive or negative) and turnaround period (long or short) were identified as key factors influencing well-being.

*I see this so much for young kids, even on our side, when you win a game on the weekend. The first thing they do is come into the change rooms and get on their phones, see all the messages that they've had* – **Player 16 (high experience)**

However, players in the present study suggested a range of solutions to combat the negative feelings, such as the introduction of charity work, off-season workshops based on mindfulness, social media education, having better contact/relationships with well-being officers, and spending time with family and friends. 

## Discussion

This study explored the perceptions of NRL players and staff on the annual training and competition calendar via a sequential explanatory design. Primary findings of this study suggest that most NRL players do not believe they are playing too many games per season and mostly disagree that the current number of games negatively impacts performance and physical or mental health when they are fit. As such, the current structure appears suitable; however, some concerns were raised, and if addressed, may optimise the well-being of all players. In particular, phase one identified that family status and player level affected well-being-related questions. Phase two highlighted that mid-year representative duties appear to have a considerable physical and mental toll on players, who appear to be affected by ‘post-origin fatigue’. Both phases revealed that players also suggest lengthening the off-season by ~ 2–4 weeks, which would optimise recovery between seasons and negate late-season fatigue, though staff believe the current schedule is appropriate. Regarding the off-season, players and staff believe this is sufficient to recover physically and mentally. However, it could be extended to allow for further mental freshness. Further, players and staff believe that the pre-season is adequate to prepare for the upcoming season and are open to reducing from 14- to 16 weeks down to approximately 12- to 14 weeks. The results of this study provide a novel overview of player and staff perceptions of the current NRL annual training and competition calendar. Findings may be used to facilitate discussion on the optimal structure of the NRL annual calendar.

Throughout the interviews, players and staff suggested that 20 to 24 games during the home-and-away season is achievable. However, some players suggested they are at their upper limit. This aligns with the survey's findings in phase one and Gouttebarge et al. [4], where 27% of NRL players and 33% of premier European footballers believe they are playing too many games each season. Phase one suggests staff perceive the number of games to negatively influence player performance (54%), physical (57%) and mental health (57%). Since the staff are responsible for monitoring and managing players during the season, their perceptions are important and contextualise player opinions. Notably, the interviews provide further context, suggesting that players often play through an injury, which is challenging to manage and affects performance within the season. However, injuries are often uncontrollable and may occur due to direct or in-direct mechanisms during training or games [29]. Regardless, incidence of match-play injuries is shown to increase across the season [29], which is consistent with phase two findings where staff suggested players are “hanging on” during the later stages of the season. Such results may argue a case for shortening the season and reducing the number of games.

In phase one, a considerable number of players agreed that the number of games played each year negatively affects their performance (25%), physical health (46%) and mental health (34%). Single players with dependants, de facto or married players with dependants, and representative players, were associated with higher frequencies of agreement regarding playing too many games. Further, single players with dependants were associated with greater agreement that the number of games negatively impacted their mental health. Despite a large proportion of players believing they are unaffected by the number of games, the NRL and associated clubs may consider providing additional support to players with dependants and their families considering that ~ 31% of players in the present study have dependants, and ~ 34% of players partake in representative games. As such, players with dependants and their families may benefit from additional support. Mental health literacy programs provided to players and performance staff could be extended to their families to develop their capacity to identify symptoms and encourage help-seeking [30, 31]. Taken together, results from both phases suggest that most players are comfortable with the current scheduling if fit. However, specific demographics within the NRL may require additional welfare support to ensure longevity in their playing careers.

Some suggestions that players and staff provided to optimise the competition structure to combat such issues were the removal of 5-day turnarounds and the inclusion of an additional bye round (weekend off). In elite Australian Rules Football, elite footballers reported improved fatigue, muscle strain, and well-being ratings following reduced training load and no competitive game [32]. As such, competition breaks within the season may have physical and psychological benefits, and periodically unloading football players might improve their performance and well-being during prolonged seasons. These breaks appear particularly important for representative players (International or State of Origin). Further, an extra bye round may be beneficial for players who are playing through minor injuries, an issue identified by both players and staff in phase two. The results of the survey suggest that players who compete in congested mid-season games generally do not perceive the additional games negatively affecting their performance or physical or mental health. However, State of Origin players were associated with a higher proportion of agreement concerning playing too many games per season than players who do not. As such, representative players might not associate the additional games to have immediate consequences, but when reflecting on the season in total, they may underestimate the negative effect of accumulative games. This is consistent with staff, who mostly agreed that mid-season representative games negatively affect players. When interviewed, players and staff suggest these games take a considerable physical and mental toll, as it is common for such players to play representative games followed by another game 2- to 4-days later. This is consistent with McLean et al. [11], who reported that neuromuscular fatigue, indicated by a countermovement jump, may take up to four days to return to baseline following a game of RL. With representative players and staff in the present study suggesting it was difficult physically and mentally, perhaps the NRL could consider legislated breaks to allow players to recover without the external pressure their respective clubs apply. Further, the State of Origin period could be played at the end of the season, as the COVID-19-impacted 2020 season re-scheduled. However, lengthening the season for such players will require a delicate rebalance of the off-season and pre-season period, accordingly.

Findings in phase two suggest the length of the off-season period was an issue for players, and most players favoured extending the off-season period to approximately 8- to 10 weeks to allow for complete mental recovery. This is consistent with the findings of phase one, where surveyed NRL players suggested that the optimal off-season length is 9.0 ± 3.8 weeks, inclusive of 3.1 ± 2.0 weeks of complete inactivity. The importance of off-season recovery for professional footballers was stressed by Gouttebarge et al. [4], who suggest that time off to regenerate between seasons is essential. When interviewed, players suggested that implementing off-season goals, such as skin folds, running times, and strength targets, could be beneficial. Some players were aware this might be a challenge for inexperienced and/or players who lacked self-motivation and would likely involve greater control of players from staff, which may negate mental recovery throughout this period. Goal setting, particularly when being monitored externally, is an effective method to increase behavioural changes [33]. No evidence supports this in elite sports; however, it may benefit player actions during the off-season. Future research in elite football could investigate adherence to off-season programs and targets to determine whether this is an effective method elite sporting clubs could employ.

Regarding the length and structure of the pre-season period, when interviewed, players strongly agreed that there could be a reduction to approximately 10- to 12 weeks. This is slightly longer than reported in phase one, where NRL players reported an optimal length was 9.2 ± 3.1 weeks. Research in elite rugby union has examined the magnitude of improvement achievable over a reduced pre-season period of 4 weeks [34]. Thirty-three elite rugby union players participated in a concurrent high-intensity aerobic, anaerobic, and resistance training block, as well as rugby-specific (tactical/technical) training. Increases in 1RM bench press (11.1 ± 2.3%), 1RM box squat (11.3 ± 4.7%) and fat-free mass (2.2% + 0.6%) were observed, whilst achieving a decrease in the sum of eight skinfolds (− 11.5 ± 2.6%) and fat mass (− 9.5 ± 2.8%). Results suggest moderate strength and fat-free mass improvements, and decreases in body fat can be achieved in a 4-week concurrent training block [34]. This suggests a shortened pre-season did not hinder elite rugby players’ physical capacities. As such, the NRL could consider exploring a reduced pre-season, using player and staff perceptual results to facilitate discussion on optimal structural changes. A common suggestion in phase two was implementing staggered start dates to reward players who adhered to exercise programs in the off-season. This could be combined with suggestions to reward players for achieving proposed off-season targets, where players who reach their goals are allowed an additional two-week break. Potentially, by providing an extrinsic reward, such as a delayed start date, professional footballers may be more inclined to arrive to pre-season in better condition [35]. Further, the idea of a progressive increase in training workload was suggested by both players and staff, who proposed returning to training part-time for the first couple of weeks (3 sessions/week).

An issue amongst players identified in phase two was the amount of contact players were exposed to during the pre-season period, raising their concerns regarding physical health and the longevity of their careers. Recently, World Rugby announced guidelines designed to reduce the contact training rugby union players undertake [36]. The guidelines suggest that, per week, players should only complete 15 min of full-contact training, during which players are unrestrained 40 min of controlled contact utilising tackle shields and pads and 30 min of live set-piece training with lineouts, scrums, and mauls. Recent research has suggested that rugby union players may experience a decline in cerebral haemodynamic function (a reduction in blood flow to the brain) and associated cognitive function due to repetitive contact and concussion [37]. Similarly, the American National Football League have imposed restrictions around contact during the pre-season, restricting contact for the first 3-weeks of the preseason. Restrictions similar to the Rugby Football Union (England) and National Football League (United States) could be considered by the NRL as a means to reduce player concerns.

The interviews highlighted many factors that influence player well-being. Most factors are uncontrollable or inevitable for players, such as match outcomes, injuries, and negative media. Highlighted factors in the present study, such as turnaround periods or overtraining, warrant control by the NRL or respective clubs. The findings regarding mental health are consistent with previous research in players that suggests game outcome is associated with short-term well-being declines [38]. As such, it is important for coaches, staff, and particularly team psychologists to appropriately screen and provide support services after a bad personal performance. Research in elite soccer (premier Portuguese competition) also reported that 64% of first-division players suggested that media strongly influenced their state of mind and felt pressured and discouraged [39]. Fazenda, Costa [39] reported that athletes self-identified solutions were to ignore and disregard negative media and try to forget what they read. Players reported that following negative reports, they tried to focus on the coach’s feedback and social support. The results from Fazenda, Costa [39] stress the importance of incorporating psychological strategies to help players deal with media pressure. For example, following negative reports, club coaches could offer positive feedback to targeted players. Additionally, player networks could be contacted for further support. Regardless, mental health literacy programs should be prioritised and provided to players and staff to help create a culture that values enhancing mental health [30]. A basic program could include athlete-specific and general mental health risk, key signs or symptoms of impaired well-being, how to seek help, basic techniques for athletes to self-manage transient mood (e.g., relaxation techniques, and mindfulness) [30]. A common theme amongst well-being discussions stressed the importance of having personal contact and a good relationship with well-being officers, and whether well-being apps were implemented as a matter of process where the outcomes are not analysed in detail. As such, individualised, routine and face-to-face mental health screening could be incorporated into a comprehensive framework monitoring player well-being within clubs.

It is important to recognise that when discussing league scheduling and broadcast negotiations, the financial aspects, and potential flow-on effects. In the NRL, broadcast rights are negotiated centrally, the revenues of which are shared equally among all clubs [40]. As such, clubs are invested in not only the strategic outcomes, but the financial outcomes achieved by league management [41]. A number of factors contribute to the negotiation power by leagues, such as exclusivity of sport (broadcast timeslots and geographic reach), supporters, and subscriptions vs. free-to-air opportunities [41]. And whilst such factors will continue to drive negotiations in the future, the present paper suggests that players well-being should also be considered during such discussions. However, clubs and their players should be aware that restructuring components of the match calendar may either positively or negatively influence revenue streams [41].

This study is not without limitations. Regarding both phases of this study, findings only apply to the NRL and potentially RL as a sport. Other organisations or football codes could conduct similar research when considering the annual training and match calendar. Further, despite the anonymity of the survey being highlighted to the participants, the NRL and RLPA endorsed this project, which may have influenced player and staff responses. Participants, either consciously or unconsciously may have responded with what the NRL or RLPA wanted to hear. Regarding phase one, a limitation of the survey is the time of the year the survey was completed (i.e., the final third of the home and away season) may have influenced responses. Reassuringly, a Chi-square test of linear trends suggests that the time surveys were completed and did not affect responses. Secondly, the sample size for staff is a potential limitation of this phase. Additional staff responses may have provided a greater depth of analysis between player and staff differences and may have allowed specific staff roles to be analysed individually.

With respect to phase two, several limitations exist. Primarily, despite sampling a range of experience, it is possible that the players who volunteered for phase two do not necessarily reflect the entire league and may better handle the workloads they are exposed to. Secondly, no validated instruments were used to assess physical and mental health, fatigue or performance, and as such, all results presented in this study are expressions of opinion and conclusions must be made with caution. Additionally, interviews were conducted following a season interrupted by COVID-19. A preamble was delivered to participants prior to the interview that directed players and staff to consider their answers for an uninterrupted training and match calendar. However, it is possible that the recent memories of a COVID-19-interrupted season skewed participants’ opinions, particularly around the lengths of each season component. Future research would benefit from contextualising perceptual data and monitoring players throughout the off-season, pre-season and in-season, using objective and valid measures of performance, fatigue, and well-being. This may provide an opportunity to contextualise perceptual research. This may result in a greater understanding of how the annual training and match calendar affects players’ physical and mental health, and further facilitate discussions around the optimal structure of the annual calendar.

## Conclusion

This study was the first to employ a mixed-methods approach to investigate the opinions of elite NRL players and staff on the NRL's annual training and match calendar. In conclusion, players and staff appear particularly comfortable with the current number of games. However, players and staff identified various structural changes and important considerations for the NRL consider in attempt to optimise the league and player well-being. Further, the results suggest specific minority groups, such as mid-year representative players and those with families, who may require additional support. Players and staff were both in favour of lengthening the off-season to allow greater recovery, shortening the pre-season, reducing contact, and implementing staggered start dates and a progressive increase in training workload. Further, structural changes, such as an additional bye round and standalone mid-season representative games, could be considered. The results of this study convey important implications for the NRL, emphasising a need to review their annual training and competitive calendar. The findings from this study should be considered when discussing the ideal length and structure of the match calendar to support players’ physical and mental welfare.

## Supplementary Information

**Additional file 1.** Survey.

**Additional file 2.** Interview schedule.

## Data Availability

As per agreement with the organisation, the datasets generated and/or analysed during the current study are not publicly available due to the sensitivity of responses.
