# Supplementary material for: The Perceptions of Elite Professional Rugby League Players and Staff on the National Rugby League Annual Calendar: A Mixed-Methods Study
Source: Sports Med Open. 2023 Jun 13;9:45. doi: 10.1186/s40798-023-00586-4 (PMC10262109; doi:10.1186/s40798-023-00586-4)
Supplement: Supplementary file 1 — Additional file 1. Survey. [file 40798_2023_586_MOESM1_ESM.docx]

**Appendix 1**. Survey

**PLAYER SURVEY**

**DEMOGRAPHICS**

Age .......................

| **What is your marital status?** | |
| --- | --- |
|  | 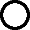 Single |
| 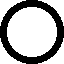 De facto | |
| 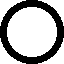 Married | |
| 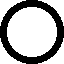 Divorced | |
| **Do you have any dependants?** | |
|  | - Yes |
| 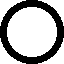 No | |

**If yes, how many?**.............

| **Primary playing position:** | |
| --- | --- |
|  | - Full-Back (1) |
| 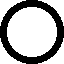 Wing (2,5) | |
| 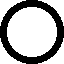 Centre (3,4) | |
| 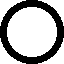 Half (6,7) | |
| 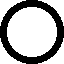 Middle Forward (8,10,13) | |
| 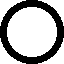 Edge Backrow (11,12) | |
| 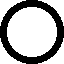 Hooker (9) | |

**Total number of first grade NRL matches**: ...........................

**Total number of NRL seasons:** ..............................................

| **Are you a Development Squad player?** | |
| --- | --- |
|  | - Yes |
| 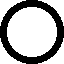 No | |
| **Have you played State of Origin in the past 5 years?** | |
|  | - Yes |
| 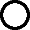 No | |
| **Have you played a Senior International match in the past 5 years?** | |
|  | - Yes |
| 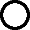 No | |

**Which Nation/s did you represent?** ...................................................................................

**IN-SEASON QUESTIONS**

| **1. I am playing too many official/competition matches (including both club and representative matches) per season:** | | | | | | |
| --- | --- | --- | --- | --- | --- | --- |
|  | Strongly Disagree | Disagree | Agree | | Strongly Agree | |
|  | 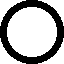 | 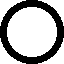 | 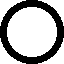 | | 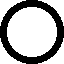 | |
| **2. The number of official/competition matches (club and representative) per season I have to play has negative consequences on my:** | | | | | | |
|  | Strongly Disagree | Disagree | Agree | | Strongly Agree | |
| a. Performance | 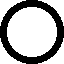 | 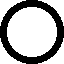 | 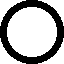 | | 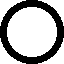 | |
| b. Physical Health | 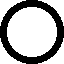 | 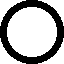 | 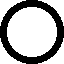 | | 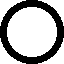 | |
| c. Mental Health | 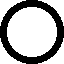 | 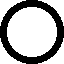 | 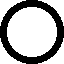 | | 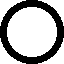 | |
| **3. Mid-year representative duties has negative consequences on my:** | | | |  | |  |
|  | Strongly Disagree | Disagree | Agree | | Strongly Agree | |
| a. Performance | 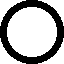 | 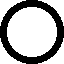 | 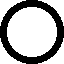 | | 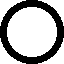 | |
| b. Physical Health | 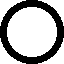 | 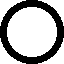 | 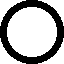 | | 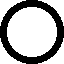 | |
| c. Mental Health | 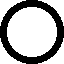 | 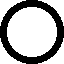 | 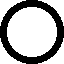 | | 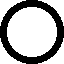 | |

| **3. End-of-year representative duties has negative consequences on my:** | | | | |  |
| --- | --- | --- | --- | --- | --- |
|  | Strongly Disagree | Disagree | Agree | Strongly Agree | |
| a. Performance | 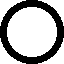 | 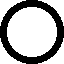 | 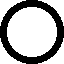 | 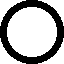 | |
| b. Physical Health | 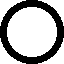 | 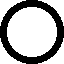 | 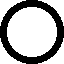 | 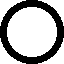 | |
| c. Mental Health | 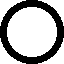 | 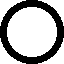 | 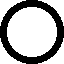 | 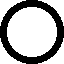 | |
| **4. In previous seasons, I have experienced moments of on-going fatigue that has negatively impacted my performance:** | | | | | |
| **a.** | Strongly Disagree | Disagree | Agree | Strongly Agree | |
|  | 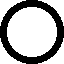 | 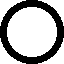 | 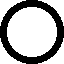 | 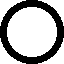 | |
| **b. At approximately what round would this on-going fatigue occur?** .................  **c. Approximately how many weeks does this fatigue last?** ................. weeks | | | | | |

**OFF-SEASON QUESTIONS**

| **5. Have you played in an NRL finals series before?** | | | | | |
| --- | --- | --- | --- | --- | --- |
| 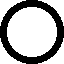 | Yes | | | | |
| 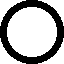 No | | | | | |
| **6. When I DO play in the finals series, the time period between the final game of the season, and the first session of pre-season, is enough recover:** | | | | | |
|  | | Strongly Disagree | Disagree | Agree | Strongly Agree |
| a. Physically | | 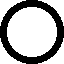 | 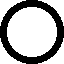 | 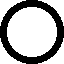 | 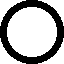 |
| b. Mentally | | 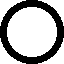 | 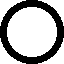 | 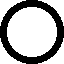 | 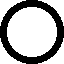 |
| **7. Have you experienced a season where you did not play in the NRL finals series?** | | | | | |
| 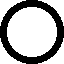 | Yes | | | | |
| 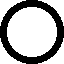 No | | | | | |
| **8. When I DO NOT play in the finals series, the time period between the final game of the season, and the first session of pre-season, is enough to recover:** | | | | | |
|  | | Strongly Disagree | Disagree | Agree | Strongly Agree |
| a. Physically | | 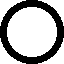 | 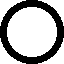 | 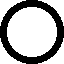 | 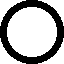 |
| b. Mentally | | 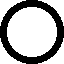 | 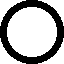 | 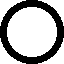 | 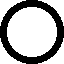 |

**9a. How many weeks following the final game of the season would you spend being completely inactive?** ................. weeks

**9b. What do you think is the optimal length of the off-season to recover both physically and mentally?** ................. weeks

**PRE-SEASON QUESTIONS**

| **10. The pre-season period (including trial games) allows sufficient time to prepare for the up-coming season:** | | | | |
| --- | --- | --- | --- | --- |
|  | Strongly Disagree | Disagree | Agree | Strongly Agree |
| a. Physically | 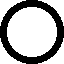 | 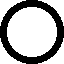 | 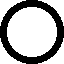 | 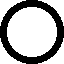 |
| b. Mentally | 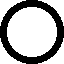 | 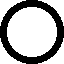 | 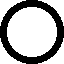 | 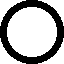 |

**11a. What do you think is the optimal length of the pre-season (in weeks), including trial games, to prepare for an upcoming season?** ................. weeks

**11b. What is the minimum number of weeks, including trial games, it would take you to prepare for an upcoming season?** ................. weeks

**END OF PLAYER SURVEY**

**STAFF SURVEY**

**DEMOGRAPHICS**

| Gender: | |
| --- | --- |
| 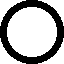 | Male |
| 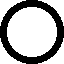 Female | |
| 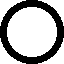 Other | |
| 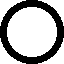 I do not want to disclose | |
| Age…………. | |
|  | |
| What is your staff role?....................................... | |
|  | |
| How many years experience do you have in this role?................. | |
| How many years experience do you have in the NRL?.................. | |

**IN-SEASON QUESTIONS**

| **Players are playing too many official/competition matches (including both club and representative matches) per season:** | | | | | | |
| --- | --- | --- | --- | --- | --- | --- |
|  | Strongly Disagree | Disagree | Agree | | Strongly Agree | |
|  | 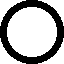 | 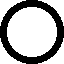 | 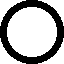 | | 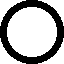 | |
| **The number of official/competition matches (club and representative) per season players play has negative consequences on their:** | | | | | | |
|  | Strongly Disagree | Disagree | Agree | | Strongly Agree | |
| Performance | 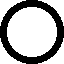 | 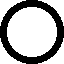 | 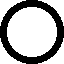 | | 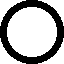 | |
| Physical Health | 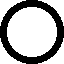 | 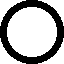 |  | |  | |
| Mental Health |  |  |  | |  | |
| **Mid-year representative duties has negative consequences on players:** | | | |  | |  |
|  | Strongly Disagree | Disagree | Agree | | Strongly Agree | |
| Performance |  |  |  | |  | |
| Physical Health |  |  |  | |  | |
| Mental Health |  |  |  | |  | |
| **End-of-year representative duties has negative consequences on players:** | | | | | |  |
|  | Strongly Disagree | Disagree | Agree | | Strongly Agree | |
| Performance |  |  |  | |  | |
| Physical Health |  |  |  | |  | |
| Mental Health |  |  |  | |  | |
| **In previous seasons, players have experienced moments of on-going fatigue that have negatively impacted their performance:** | | | | | | |
|  | Strongly Disagree | Disagree | Agree | | Strongly Agree | |
|  |  |  |  | |  | |

**At approximately what round would this on-going fatigue occur?** .................

**Approximately how many weeks does this fatigue last?** ................. weeks

**OFF-SEASON QUESTIONS**

| **Have you experienced a season where your associated team played in the NRL finals series?** | | | | | |
| --- | --- | --- | --- | --- | --- |
|  | Yes | | | | |
| No | | | | | |
| **When players DO play in the finals series, the time period between the final game of the season, and the first session of pre-season, is enough for them to recover:** | | | | | |
|  | | Strongly Disagree | Disagree | Agree | Strongly Agree |
| Physically | |  |  |  |  |
| Mentally | |  |  |  |  |
| **Have you experienced a season where your associated team did not play in the NRL finals series?** | | | | | |
|  | Yes | | | | |
| No | | | | | |
| **When players DO NOT play in the finals series, the time period between the final game of the season, and the first session of pre-season, is enough for them to recover:** | | | | | |
|  | | Strongly Disagree | Disagree | Agree | Strongly Agree |
| Physically | |  |  |  |  |
| Mentally | |  |  |  |  |

**What do you think is the optimal length of the off-season (in weeks) for players to recover from the season both physically and mentally?** ................. weeks

**PRE-SEASON QUESTIONS**

| **The pre-season period (including trial games) allows sufficient time to prepare players for the up-coming season:** | | | | |
| --- | --- | --- | --- | --- |
|  | Strongly Disagree | Disagree | Agree | Strongly Agree |
| Physically |  |  |  |  |
| Mentally |  |  |  |  |

**What do you think is the optimal length of the pre-season, including trial games, to prepare players for the upcoming season?** ................. weeks

**What is the minimum number of weeks, including trial games, it would take you to prepare players for an upcoming season?** ................. weeks

**END OF STAFF SURVEY**
