# Supplementary material for: The Perceptions of Elite Professional Rugby League Players and Staff on the National Rugby League Annual Calendar: A Mixed-Methods Study
Source: Sports Med Open. 2023 Jun 13;9:45. doi: 10.1186/s40798-023-00586-4 (PMC10262109; doi:10.1186/s40798-023-00586-4)
Supplement: Supplementary file 2 — Additional file 2. Interview schedule. [file 40798_2023_586_MOESM2_ESM.docx]

**Off-season and Pre-season questions**

**Q1. What do you think is the optimal period for the off-season?** Please explain.

What is important to consider to get this period right (e.g. for players, football staff, the club)?

What are some of challenges or constraints, in this regard?

How do you feel as off-season is finishing and you’re about to enter pre-season?

- Mentally
- Physically

**Q2. What do you think is the optimal period for the pre-season?** Please explain.

How do you think the pre-season should be structured?

- Any specific training blocks?
- Are there specific skills or physical sessions that require more or less attention?

What is important to consider to get this period right (e.g. for players, football staff, the club)?

- What are some of challenges or constraints, in this regard?
- Is there a point in the pre-season where you start to feel match fit?
- How do you feel (mentally and physically) as pre-season is finishing and you’re about to enter the competition?
  - Compared to the start of pre-season?

**In-season/Match Calendar questions**

**Q3. Typically, how many games should NRL players be playing every season (both club and representative levels)?** Please explain.

What impact is the current number of games having on you?

- Performance level
- Physical health
- Mental health

**Q4. What changes, if any, should be made in game scheduling?**

How do you feel about week-to-week recovery?

**Q5. For representative athletes, how do you think representative games affect you?**

- Performance level
- Physical health
- Mental health

**Performance: Staff focus**

**Q6. What factors contribute to player fatigue during the season?**

- Positive factors
- Negative factors

**Q7. What factors contribute to player performance levels during the season?**

- Positive factors
- Negative factors

**Q8. How does the club currently monitor player workloads during the season?**

How successful is this process at managing player fatigue (mental and physical)?

How successful is this process to maintaining player performance levels?

**Q9. What changes (if any) need to occur to improve the process of managing player fatigue / maintaining player performance levels in-season?**

What key metrics should be monitored? How should this information be best used?

**Wellbeing: Athlete focus**

**Q10. How does the club currently monitor player wellbeing during the season?**

Currently, how successful is this process?

What changes (if any) need to occur to improve the monitoring and management of player wellbeing?

**Q11. What factors impact player wellbeing (physical / mental / emotional health) during the season?**

- Positive factors
- Negative factors
